# Infection markers as predictors of Bacteremia in an Intensive Care Unit: A prospective study

**DOI:** 10.12669/pjms.346.15665

**Published:** 2018

**Authors:** Pinar Sen, Tuna Demirdal, Salih Atakan Nemli, Ilknur Vardar, Mehmet Kizilkaya, Atilla Sencan, Huriye Erbak Yilmaz

**Affiliations:** 1 *Pinar Sen, M.D. Department of Infectious Diseases and Clinical Microbiology Izmir Katip Celebi University Ataturk Training and Research Hospital, Katip Celebi University Ataturk Research and Training Hospital 35360, Karabaglar, Izmir, Turkey*; 2 *Prof. Tuna Demirdal, Department of Infectious Diseases and Clinical Microbiology Izmir Katip Celebi University Ataturk Training and Research Hospital, Katip Celebi University Ataturk Research and Training Hospital 35360, Karabaglar, Izmir, Turkey*; 3 *Salih Atakan Nemli, M.D., Department of Infectious Diseases and Clinical Microbiology Izmir Katip Celebi University Ataturk Training and Research Hospital, Katip Celebi University Ataturk Research and Training Hospital 35360, Karabaglar, Izmir, Turkey*; 4 *Ilknur Vardar; M.D., Department of Infectious Diseases and Clinical Microbiology Izmir Katip Celebi University Ataturk Training and Research Hospital, Katip Celebi University Ataturk Research and Training Hospital 35360, Karabaglar, Izmir, Turkey*; 5 *Prof. Mehmet Kizilkaya, Department of Anesthesiology and Reanimation, Izmir Katip Celebi University Ataturk Training and Research Hospital, Katip Celebi University Ataturk Research and Training Hospital 35360, Karabaglar, Izmir, Turkey*; 6 *Atilla Sencan, M.D, Department of Anesthesiology and Reanimation, Izmir Katip Celebi University Ataturk Training and Research Hospital, Katip Celebi University Ataturk Research and Training Hospital 35360, Karabaglar, Izmir, Turkey*; 7 *Huriye Erbak Yilmaz, MD. Department of Biochemistry, Izmir Katip Celebi University Ataturk Training and Research Hospital, Katip Celebi University Ataturk Research and Training Hospital 35360, Karabaglar, Izmir, Turkey*

**Keywords:** Bacteremia, Intensive care unit, Neopterin, Neutrophil to lymphocyte ratio, Pro-adrenomedullin

## Abstract

**Objective:**

Although several biomarkers have been evaluated for the diagnosis and prognosis of sepsis, the gold standard biomarker has not yet been found. We aimed to evaluate the diagnostic value of neutrophil-to-lymphocyte count ratio (NLCR), neopterin, pro-adrenomedullin (pro-ADM) and the other infection markers to predict bacteremia in patients with SIRS, sepsis and severe sepsis/septic shock.

**Methods:**

A prospective cohort study was conducted on septic patients in a tertiary referral hospital between December 2014- July 2015. A total of 156 patients diagnosed with SIRS, sepsis and severe sepsis/septic shock in Anesthesia intensive care unit (ICU) were included in the study.

**Results:**

A total of 156 patients who had been diagnosed as SIRS(10.9%), sepsis (44.2%) and severe sepsis/septic shock (44.9%) were included. Positive blood cultures were obtained in 64 patients. NLCR, neopterin and pro-ADM levels were insignificant in predicting bacteremia (p>0.05). The mortality rate was significantly higher in bacteremic sepsis (43.9%) compared to non-bacteremic patients (20.8%) (p=0.001). Only procalcitonin levels were significant predictor of mortality (p<0.001).

**Conclusion:**

NLCR, CRP, procalcitonin, neopterin and pro-ADM levels were insignificant in diagnosis of bacteremia in critically ill patients. The gold standard method in predicting bacteremia is still blood culture positivity.

## INTRODUCTION

Sepsis is defined as uncontrolled immune response against the infections and common health problem with increasing incidence in the last two decades.^1^ It has been reported that more than 650,000 people are affected each year by sepsis.^2^ Despite all advances in medicine, it remains a serious clinical problem with an overall mortality of about 30%.^3^,^4^ In recent years, incidence and mortality rate of sepsis has increased rapidly due to invasive procedures and aggressive treatments especially in intensive care units (ICU).^5^

The gold standard for the diagnosis of sepsis is the presence of infection with the isolation of microorganisms in blood culture.^6^,^7^ Early diagnosis and treatment is very important to improve survival in sepsis.^3^,^8^ However microorganisms can be detected in 30% of blood cultures and it usually takes 48 to 72 hours.^9^ Biomarkers have a significant role in the diagnosis of sepsis. Even though inflammatory markers including C-reactive protein (CRP) and procalcitonin are useful in the diagnosis and prognosis of sepsis in clinical practice, non-infectious causes also may lead to increase in blood levels of these biomarkers.^10^,^11^ Although several biomarkers have been evaluated for the diagnosis and prognosis of sepsis, the gold standard biomarker has not yet been found.

New biomarkers; neutrophil-to-lymphocyte count ratio (NLCR), neopterin and pro-adrenomedullin (pro-ADM) have been reported in recent studies as a potential biomarkers in the diagnosis and prognosis of sepsis.^12^-^14^ These biomarkers were evaluated separately in previous studies but there is no study comparing these markers with each other in the literature.^15^,^16^ We aimed to evaluate and compare the diagnostic value of NLCR, neopterin, pro-ADM and the other infection markers in patients with systemic inflammatory response syndrome (SIRS), sepsis and severe sepsis/septic shock in ICU.

## METHODS

We conducted a prospective cohort study at the Katip Celebi University, Ataturk Training and Research Hospital, a 1055-bed tertiary referral care center contains 86-bed adult ICU in Izmir, Turkey. The study protocol was approved by The Ethics Committee of Katip Celebi University, Izmir, Turkey (Date: 10.09.2014, Decision no: 140). Informed written consent was obtained from first-degree relatives of patients. A total of 156 patients diagnosed with SIRS, sepsis and severe sepsis/septic shock in Anesthesia ICU were included between December 2014-July 2015. The study group consisted of 64 bacteremic patients and the control group was consisted of 92 non-bacteremic patients.

Exclusion criteria included age less than 18 years, contaminated blood cultures, hematologic malignancy, chemotherapy, fungemia, HIV infection and parasitic infections that increase the value of eosinophils. A blood culture contamination were evaluated if these microorganisms detected in only one culture from simultaneously taken two blood culture within 24 hours: coagulase-negative Staphylococci, *Bacillus* spp., *Propionibacterium acnes*, *Corynebacterium* spp., *Micrococcus* spp. However, determining of this organisms in both blood cultures with same sensitivity were considered as true-positive.

SCCM (Society of Critical Care Medicine) and ESICM (European Society of Intensive Care Medicine) consensus criteria (2012) were used in the diagnosis of sepsis.^1^ Blood cultures and blood samples for neopterin, pro-ADM and the other infection markers were taken during the admission before the first dose of empirical antibiotics. The NLCR was calculated using neutrophil and lymphocyte levels. Data including demographic data, vital signs, comorbidity, cause of hospitalization, length of stay in the ICU, prior antibiotic use, mechanical ventilation, source of infection, in-hospital mortality rates, APACHE II, SOFA and Charlson comorbidity index scores were recorded. Laboratory parameters, vital signs and scoring systems were based on maximum deviation from normal values and physiologic variables within 24 hours of admission to the ICU. Statistical analysis was performed to determine the value of the NLCR and other infection markers for predicting bacteremia between study and control groups.

### Measurement of blood samples

Positive blood cultures were identified by using the BACTEC FX automatic blood culture detection system (Becton Dickinson, Sparks, MD, USA) in the medical microbiology laboratory. The antimicrobial susceptibility of isolated strains has been determined by Phoenix Automated Microbiology System (BD Diagnostic Systems, Sparks, MD) in accordance with EUCAST (The European Committee on Antimicrobial Susceptibility Testing) criteria.

Venous blood samples (8-10 ml) were obtained simultaneously with blood cultures to determine the level of the neopterin and pro-ADM. Blood samples were collected in serum separator tubes under sterile conditions and centrifuged at 3000 rpm for 15 minutes. Serum samples were stored at -80°C freezer in Eppendorf tubes until the day of analysis. Hemolyzed or lipemic specimens were excluded from the study. Samples were thawed and analyzed via ELISA on the day of study.

Neopterin ELISA kit (YH-biosearch, Shangai, China, reference no: YHB20150518449, YHB20150706516; lot no: 20150518, 20150706) and pro-ADM ELISA kit (YH-biosearch, Shangai, China, reference no: YHB20150518448, YHB20150706515; lot no: 20150518, 20150706) was used and analyzed on a semiautomatic ELISA analyzer. The absorbance was measured at 450 nm by an enzyme micro-plate reader (Bio Tek, ELX 800, USA).

### Statistical analyses

Statistical analysis of the data were performed in two groups consisting of bacteremic and non-bacteremic patients for predicting bacteremia and mortality using SPSS (version 22) software (IBM SPSS, USA) package with 95% confidence. Chi-square test, Fisher’s exact chi-square test, trend test and Mann Whitney U test was used for the comparison of categorical data between the groups. The significance of NLCR, neopterin and pro-ADM levels on predicting bacteremia was evaluated by receiver operating characteristic (ROC) curve analysis. Survival data of the patients were evaluated by Kaplan-Meier survival analysis. P value <0.05 was considered statistically significant.

## RESULTS

**Fig.1:**
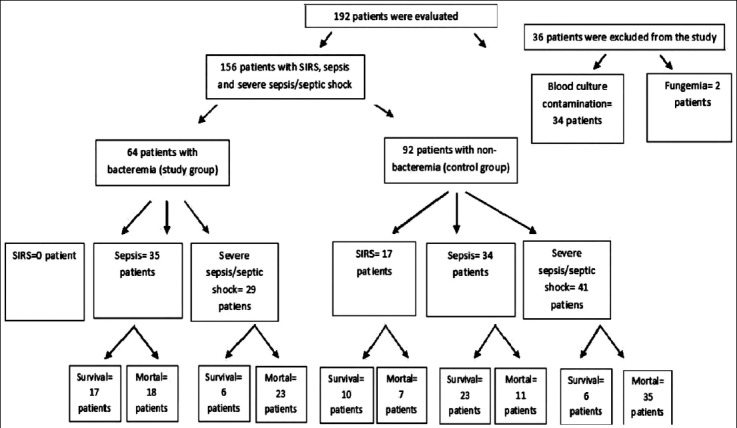
Flow chart of the study.

A total of 192 patients admitted to the Anesthesia ICU diagnosed as SIRS, sepsis and severe sepsis/septic shock were evaluated prospectively during the 2014-2015 study period. Among these patients, 36 (15.8%) patients were excluded from the study due to fungemia (n=2, 0.8%) and blood culture contamination (n=34, 15%) (Fig. 1). One hundred fifty six patients who had been diagnosed as SIRS (n=17, 10.9%), sepsis (n=69, 44.2%), and severe sepsis/septic shock (n=70, 44.9%) were enrolled. The study group consisted of 64 (41%) bacteremic patients. The control group consisted of 92 (59%) non-bacteremic patients. The mean age ± standart deviation (SD) was 60.5±16.7 years. Of these patients, 99 (63.5%) were male. Demographic data and clinical findings of the study and control groups are shown in Table-I.

**Table-I:** Demographic and clinical characteristics of bacteremic and non-bacteremic groups.

	Bacteremic group n=64	Non-bacteremic group n=92	P-value
Male (n, %)	41(26.3)	58(37.2)	
Female (n, %)	23(14.7)	34(21.8)	
Age (years, mean±SD)	59.8±17.6	61±16.1	0.837
Smoking (n,%)	14(36.8)	24(63.2)	0.547
Alcohol use (n,%)	3(37.5)	5(62.5)	1.000
History of antibiotic use (n, %)	26(16.7)	32(20.6)	
Length of stay (days, mean±SD)	39.8±54.7	17.5±34.6	0.015
***Diagnoses (n,%)***			
SIRS	-	17(18.5)	
Sepsis	35(54.7)	34(37.0)	0.028
Severe sepsis / Septicshock	29(45.3)	41(44.6)	0.926
***Comorbidity (n,%)***			
Diabetes mellitus	19(29.7)	24(26.1)	0.621
Chronic heart failure	6(9.4)	12(13.0)	0.481
Hypertension	20(31.3)	33(35.9)	0.549
Chronic renal disease	10(15.6)	11(12.0)	0.549
Solid organ malignancy	5(7.8)	8(8.7)	0.844
Chronic lung disease	5(7.8)	22(23.9)	0.009
Cardiac arrhythmia	2(3.1)	11(12.0)	0.049
Coronary heart disease	8(12.5)	17(18.5)	0.317
Cerebrovascular disease	8(12.5)	8(8.7)	0.441
***Management (n,%)***			
Medical patients	37(57.8)	51(55.4)	0.768
Surgical patients	27(42.2)	41(44.6)	
Mechanical ventilation	48(75.0)	76(82.6)	0.247
Vasopressor use	29(45.3)	41(44.6)	0.926
***Laboratory findings (n, mean±SD)***			
White blood cell count (K/uL)	13169.4±7427.0	15404.8±9105.5	0.133
Hemoglobin (g/dL)	10.0±1.7	10.4±2.1	0.157
Eosinophil (K/uL)	118.1±201.9	72.9±110.6	0,181
Platelet (K/uL)	206578.1±135123.6	241608.7±143198,3	0.141
Sedimentation (mm)	59.9±31.1	57.1±33.5	0.551
CRP (mg/dl)	18.6±8.9	17.5±10.1	0.343
PCT (ng/dl)	11.9±21.5	5.9±11.5	0.168
NLCR	14.9±15.2	16.6±15.0	0.337
Neopterin (nmol/L)	20.9±36.2	12.1±13.9	0.167
Pro-ADM (ng/L)	632.0±721.0	438.2±393.1	0.172
***Mortality (n,%)***			
Overall mortality	41(43.6)	53(56.4)	0.418
28-day mortality	29(36.7)	50(63.3)	0.002
APACHEII score(mean±SD)	24.5±8.9	24.5±8.9	0.891
SOFA score(mean±SD)	9.4±4.1	9.1±4.3	0.804
Charlson comorbidity index(mean±SD)	5.4±3.5	5.4±3.6	0.830

*SD: Standart Deviation, SIRS: Systemic inflammatory response syndrome, CRP: C-reactive protein.

Gender distribution was similar in both groups, however female patients with a positive blood culture were older (64.9±18.7 years) than male patients with bacteremia (56.9±16.4 years) (p=0.023).

There were no statistically significant differences between the two groups in terms of smoking and alcohol use (p=0.547 and p=1.000, respectively).The incidence of chronic lung disease and cardiac arrhythmia in the study group was statistically higher than the control group (p=0.009, p=0.049 respectively).

Source of infections were determined as follows; lower respiratory tract infections (n=94, 60.3%), intra-abdominal infections (n=43, 27.6%), urinary tract infections (n=17, 10.9%), skin and soft tissue infections (n=16, 10.3%), catheter infections (n=5, 3.2%), central nervous system infections (n=2, 1.3%), bone and joint infections (n=1, 0.6%). Multiple focus of infection was detected in 22 (14.1%) patients.

The average length of stay in the ICU in bacteremic patients was 39.8±54.7 days and in the control group was 17.4±34.6 days. Length of stay in the ICU was found significantly higher in the presence of bacteremia (p=0.015). The 37.2% of all 156 patients had a history of prior antibiotic use.

### Laboratory Findings

Laboratory parameters, vital signs and scoring systems were not statistically different between the study and control groups (Table I).

Blood cultures were positive in 64 (28.3%) patients, 31 (48.4%) of them were gram positive, 37 (57.8%) of them were gram negative bacteria, respectively (*Escherichia coli* (n=13, 20.3%), coagulase-negative stapyhlococci (n=12, 18.8%), *Acinetobacter baumanii* (n=11, 17.2%), *Staphylococcus aureus* (n=9, 14.1%), *Klebsiella* spp. (n=4, 6.3%), *Stenotrophomonas maltophilia* (n=3, 4.7%), *Enterococcus faecalis* (n=3, 4.7%), *Enterococcus faecium* (n=2, 3.1%), *Gemella* spp. (n=2, 3.1%), *Pseudomonas aeroginosa* (n=1, 1.6%) and others (n=8, 12.8%)). Multiple positive blood cultures were found in 5 (7.8%) patients.

CRP, procalcitonin, neopterin and pro-ADM levels were found worthless in determining the types of bacteria (gram-negative or gram-positive) in patients with bacteremia (18.8±8.4 versus 18.4±9.6 mg/dl, p=0.662; 16.4±27.6 versus 7.3±10.7 ng/dl, p=0.145; 18.6±28.2 versus 23.5±43.4 nmol/L, p=0.409; 548.1±486.3 versus 721.3±907.4 ng/L, p=0.638, respectively). Mean CRP levels and NLCR were similar between both groups (p=0.343, p=0.337) (Table I). Likewise, procalcitonin, neopterin and pro-ADM levels were statistically insignificant in predicting bacteremia although their levels in the bacteremic group were higher than non-bacteremic group (p=0.343, p=0.168, p=0.337, p=0.167, p=0.172, respectively)

CRP, procalcitonin, neopterin and pro-ADM levels, Charlson index, APACHE II score, SOFA score was not statistically different among medical and surgical patients (p=0.151, p=0.371, p=0.752 p=0.551, p=0.518, p=0.939, p=0.817, respectively).

APACHE II scores, CRP and procalcitonin levels in patients with a diagnosis of SIRS were significantly lower compared to patients with diagnosis of sepsis and severe sepsis/septic shock (p=0.031, p=0.022, p<0.001, respectively), whereas there were no significant differences between these groups in terms of Charlson index, SOFA score, neopterin and pro-ADM levels (p=0.139, p=0.115, p=0.997, p=0.233, respectively).

**Fig.2:**
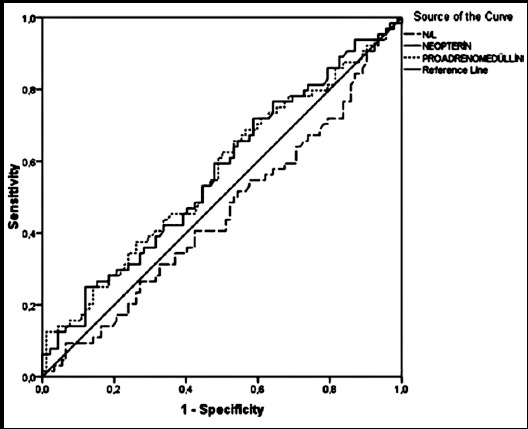
ROC curves of NLCR, neopterin and pro-ADM for predicting bacteremia.

ROC analysis demonstrated that neopterin and pro-ADM had the highest area under the curve (AUC) compared to NLCR for predicting bacteremia in ICU. AUC value for NLCR was 0.455 (95% confidence interval (CI) 0.362-0.547), neopterin was 0.565 (95% CI 0.473-0.657) and pro-ADM was 0.564 (95% CI 0.472-0.657) (Fig. 2).

The sensitivity was very low (9.4%) however high specificity (93.5%) was determined when NLCR cut-off value was calculated as 39.4. In the bacteremic group, six patients had an NLCR higher than 39.4 and six patients in non-bacteremic group. Optimal cut-off values were determined as 7.2 for neopterin (46 patients in bacteremic group and 54 patients in non-bacteremic group when neopterin was higher than 7.2) and 367.6 for pro-ADM (40 patients in bacteremic group and 46 patients in non-bacteremic group when pro-ADM was higher than 367.6), the sensitivity and specificity were as follows; 71.9%, 41.3%, 62.5%, 50.0%, respectively (Table II).

**Table-II:** Sensitivity, specificity, positive predictive value (PPV) and negative predictive value (NPV) of the NLCR, neopterin and pro-ADM in predicting bacteremia.

	Cut-off value	Sensitivity (%)	Specificity (%)	PPV (%)	NPV (%)
NLCR(n)	39.41	9.4	93.5	50.0	134.0
Neopterin(nmol/L)	7.22	71.9	41.3	46.0	59.4
Pro-ADM(ng/L)	367.60	62.5	50.0	46.5	71.9

### Mortality

The mean age of nonsurvivors (64.6±13.7 years) was significantly higher than the survivors (54.4±18.8 years) (p=0.001). On the other hand, there were no statistically significant differences between nonsurvivors (65.5±13.3 years) and survivors (59.7±16.1 years) in terms of 28 day-mortality (p=0.262).

The overall mortality rate was found 60.3% (94/156) and 50.6% (79/156) of them died within the first 28 days of follow-up. There was no significant difference between the study and control groups in terms of overall mortality (p=0.418), but 28-day mortality rate in bacteremic patients was significantly higher than the control group (p=0.002).

The mortality rate was significantly higher in bacteremic sepsis (43.9%) compared to non-bacteremic patients (20.8%) (p=0.001). In contrast to this finding, the mortality rate in bacteremic patients with severe sepsis/septic shock (56.1%) was significantly lower compared to non-bacteremic severe sepsis/septic shock patients (66.0%) (p=0.021). Only procalcitonin levels were significantly higher in nonsurvivors compared to survivors (10.1±18.0 versus 5.7±13.7 ng/dl; p<0.001).

APACHE II, SOFA and Charlson-comorbidity index scores were similar between the bacteremic and non-bacteremic groups. However, mean APACHE II score (27.9±7.6 versus 19.3±8.1), SOFA score (10.6±3.6 versus 7.2±4.2) and Charlson comorbidity index (6.0±3.1 versus 4.5±3.9) were significantly higher in nonsurvivors than survivors (p<0.001, p<0.001, p=0.006 respectively).

**Fig.3:**
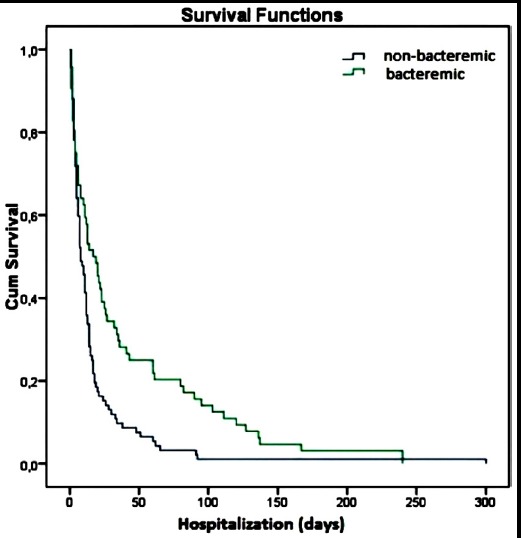
Kaplan-Meirer survival curves for the presence of bacteremia.

**Fig.4:**
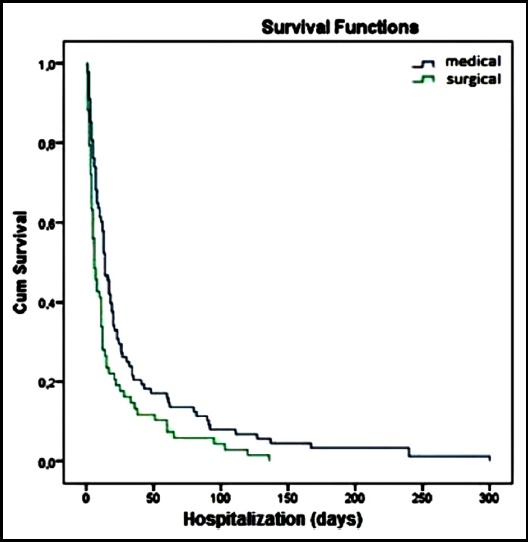
Kaplan-Meirer survival curves for the medical/surgical condition.

The median survival time was 11 days in all patients, 17 days in bacteremic patients and eight days in non-bacteremic patients, 14 days in medical patients and six days in surgery patients, 11 days in patients with NLCR value <39 and 14 days in patients with NLCR value >39. The median survival was significantly higher in bacteremic patients and medical patients (p=0.001, p=0.005 respectively) (Fig. 3,4). There was no statistically significant difference in median survival for NLCR (p=0.389).

The correlation between Charlson index and NLCR was positive but weak and the statistical difference was found significant (p=0.015).

We found a statistically significant correlation between the neopterin and CRP with APACHE II score (p=0.004, p=0.047 respectively). The correlation between CRP and APACHE II was positive. There was a weak, negative correlation between neopterin and APACHE II.

The relationship between SOFA score and biomarkers was as follows: the positive correlation between SOFA and CRP also with procalcitonin, the weak negative correlation between SOFA and neopterin also with pro-ADM. The statistical difference was statistically significant (p<0.001, p<0.001, p=0.028, p=0.012 respectively).

## DISCUSSION

Bacterial infections are the main causes of morbidity and mortality in critically ill patients.^4^ Timely and accurate diagnosis and antimicrobial treatment is mandatory to improve survival in sepsis.^3^,^8^ Blood culture positivity has been determined as the gold standard, however microorganisms can be detected in 30% of blood cultures and it usually takes 48 to 72 hours.^9^ Molecular methods give faster and more accurate results, but they are expensive and they cannot be achieved at all centers.^6^,^7^ Biomarkers have a significant role in detecting bacterial infection. Although several biomarkers have been evaluated for the diagnosis and prognosis of sepsis, the gold standard biological marker has not yet been found. In our study, NLCR, neopterin, pro-ADM and the other infection markers was investigated in septic patients as predictors of bacteremia.

In inflammatory conditions, neutrophil count increases when lymphocyte count decreases.^17^ Therefore, NLCR increases in infectious diseases and have turned into inflammatory markers. There are some studies indicating that the changes in the rate of circulating leukocytes can recognize the bacteremia at an early stage.^3^ Firstly, Zahorec and colleagues indicated that lymphocytopenia is correlated with the severity of sepsis in oncology ICU, therefore they claimed NLCR could be a marker of infection.^17^ Then Wyllie and colleagues showed the significance of NLCR in predicting bacteremia compared to lymphocytopenia or CRP.^18^ Various studies have been performed for determination of the optimal NLCR cut-off value based upon these findings, but obtained different cut-off value for sepsis and bacteremia. Loonen and colleagues reported the high sensitivity and specificity in predicting bacteremia when the cut-off value was accepted as 10.^12^ Gurol and colleagues demonstrated that optimal NLCR cut-off value should be more than five for identifying sepsis due to effects of surgery, trauma and rheumatic disease.^19^,^20^ In our study we found the higher cut-off value than the other studies. We thought the high cut off value of NLCR was found in our study as a result of the monitoring of the patients in tertiary ICU. Therefore we believe that NLCR is not a useful marker to predict bacteremia in ICU.

It is stated that the neopterin and pro-ADM is useful for detecting infection and severity of illness.^21^,^22^ The upper limit of serum neopterin level was shown as 10 nmol/L in healty individuals and it was reported that neopterin could be a useful marker in predicting infection and gram negative sepsis.^23^ In patients with sepsis, mean neopterin level was found 75.7 nmol/L and shown to be significant predictor of bacteremia.^21^ The pro-ADM cutoff value was calculated as 1 nmol/L to distinguish septic patients from healthy individuals. In particular serum levels of pro-ADM were significantly higher in patients with gram positive bacteremia and fungemia.^22^ In the present study, neopterin and pro-ADM levels were higher in bacteremic patients than the control group, although the difference was not statistically significant (p=0.167, p=0.172 respectively). Therefore, we believe that neopterin and pro-ADM levels were inadequate to indicate the disease severity and detection of infectious diseases.

CRP and procalcitonin are the most widely used markers for bacterial infections. In a study conducted in China, it was determined that the CRP and procalcitonin levels increased significantly in infectious disease.^20^ Similarly, our study also showed an increase of these markers in the presence of infection. In contrast, these two markers were found insignificant in predicting bacteremia.

It is important to determine the etiology of sepsis for treatment success in terms of reducing mortality. However, it is reported that positive blood cultures were obtained in 50-60% patients with the diagnosis of sepsis.^17^ In our study, the isolation rates of bacteria in blood culture were 28.3% in all patients. Our isolation rate is lower than the previous reports, we interpret this finding as a result of the history of antibiotic use (37.2%).

The reported mortality rates are still high despite advances in the treatment of sepsis. The overall mortality rate was declared around 80% in ICU about 30 years ago, it is stated that decreased to 20-30% in recent years.^24^ In a study conducted by Iwashyna and colleagues, the overall mortality rate was reported as 15.8% and 30-day mortality rate was 24.9% for sepsis.^5^ The mortality rate was high in our ICU, we considered that as a result of different conditions and different group of patients with various comorbidities. In addition, we also believe that lower mortality rate in bacteremic patients with severe sepsis/septic shock compared to the non-bacteremic patients is a point that deserves exploration through extensive researches.

In some recent studies, it was aimed to evaluate the easily accessible, low-cost, new prognostic marker in predicting mortality in ICU. Although shown the prognostic significance of NLCR was demonstrated in a study that included 2,311 patients, the prognostic value was not demonstrated in another study of 5056 patients analyzed.^25^ Only procalcitonin was found to be significant among the examined markers in predicting mortality in our study. Therefore, we believe that procalcitonin may be a good prognostic marker in ICU. Other cost-effective, easily accessible infection markers can be found for predicting bacteremia and mortality with large-scale studies in critically ill patients.

### Limitations of the study

Clinical criteria for sepsis were revised in 2016 by Singer M et al.^26^ However, our patients were evaluated between 2014 and 2015, therefore ESICM consensus criteria (2012) were used in the diagnosis of sepsis. Another limitation was a single center study and it did not evaluate the stabilities of these biomarkers over time.

## CONCLUSION

As a result, NLCR, CRP, procalcitonin, neopterin and pro-ADM levels were insignificant in diagnosis of bacteremia. The gold standard method in predicting bacteremia is still blood culture positivity in intensive care units.

### Author`s Contribution

**PS, TD** conceived and designed of manuscript.

**PS, TD, SAN, IV, MK, AS and HEY** did data collection and manuscript writing.

**TD** did review and final approval of manuscript.

## References

[1] Dellinger RP, Levy MM, Rhodes A, Annane D, Gerlach H, Opal SM (2013). Surviving Sepsis Campaign Guidelines Committee including The Pediatric Subgroup. Surviving Sepsis Campaign:international guidelines for management of severe sepsis and septic shock, 2012. Intensive Care Med.

[2] Okashah AS, El-Sawy MM, Beshay BN, Abd El-Raouf A (2014). Ratio of neutrophil to lymphocyte counts as a simple marker for sepsis and severe sepsis in intensive care unit. Res Opin in Anesth Intensive Care.

[3] de Jager CP, van Wijk PT, Mathoera RB, de Jongh-Leuvenink J, van der Poll T, Wever PC (2010). Lymphocytopenia and neutrophil-lymphocyte count ratio predict bacteremia better than conventional infection markers in an emergency care unit. Crit Care.

[4] Dagher GA, Saadeldine M, Bachir R, Zebian D, Chebl RB (2015). Descriptive analysis of sepsis in a developing country. Int J Emerg Med.

[5] Iwashyna TJ, Cooke CR, Wunsch H, Kahn JM (2012). Population burden of long-term survivorship after severe sepsis in older Americans. J Am Geriatr Soc.

[6] Peters RP, van Agtmael MA, Danner SA, Savelkoul PH, Vandenbroucke-Grauls CM (2004). New developments in the diagnosis of bloodstream infections. Lancet Infect Dis.

[7] Mancini N, Carletti S, Ghidoli N, Cichero P, Burioni R, Clementi M (2010). The era of molecular and other non-culture-based methods in diagnosis of sepsis. Clin Microbiol Rev.

[8] Pierrakos C, Vincent JL (2010). Sepsis biomarkers:a review. Crit Care.

[9] Wacker C, Prkno A, Brunkhorst FM, Schlattmann P (2013). Procalcitonin as a diagnostic marker for sepsis:a systematic review and meta-analysis. Lancet Infect Dis.

[10] Jeong S, Park Y, Cho Y, Kim HS (2012). Diagnostic utilities of procalcitonin and C-reactive protein for the prediction of bacteremia determined by blood culture. Clin Chim Acta.

[11] Nargis W, Ibrahim M, Ahamed BU (2014). Procalcitonin versus C-reactive protein:Usefulness as biomarker of sepsis in ICU patient. Int J Crit Illn Inj Sci.

[12] Loonen AJ, de Jager CP, Tosserams J, Kusters R, Hilbink M, Wever PC (2014). Biomarkers and molecular analysis to improve bloodstream infection diagnostics in an emergency care unit. PLoS One.

[13] Girgin G, Sahin TT, Fuchs D, Yuksel O, Kurukahvecioglu O, Sare M (2011). Tryptophan degradation and serum neopterin concentrations in intensive care unit patients. Toxicol Mech Methods.

[14] Yan L, Cailan L, Hong L, Zheng L, Wei D (2015). The value of pro-adrenomedullin in early diagnosis of sepsis. Zhonghua Wei Zhong Bing Ji Jiu Yi Xue.

[15] Lowsby R, Gomes C, Jarman I, Lisboa P, Nee PA, Vardhan M (2015). Neutrophil to lymphocyte count ratio as an early indicator of bloodstream infection in the emergency department. Emerg Med J.

[16] Dunne WM (2015). Laboratory Diagnosis of Sepsis?No SIRS, Not Just Yet. J Clin Microbiol.

[17] Zahorec R (2001). Ratio of neutrophil to lymphocyte counts—Rapid and simple parameter of systemic inflammation and stress in critically ill. Bratisl Lek Listy.

[18] Wyllie DH, Bowler IC, Peto TE (2005). Bacteraemia prediction in emergency medical admissions:role of C reactive protein. J Clin Pathol.

[19] Gürol G, Çiftci ĠH, Terizi HA, Atasoy AR, Ozbek A, Köroğlu M (2015). Are there standardized cutoff values for neutrophil-lymphocyte ratios in bacteremia or sepsis?. J Microbiol Biotechnol.

[20] Su L, Feng L, Song Q, Kang H, Zhang X, Liang Z (2013). Diagnostic value of dynamics serum sCD163, sTREM-1, PCT, and CRP in differentiating sepsis, severity assessment, and prognostic prediction. Mediators Inflamm.

[21] Sheldon J, Riches PG, Soni N, Jurges E, Gore M, Dadian G (1991). Plasma neopterin as an adjunct to C-reactive protein in assessment of infection. Clin Chem.

[22] Angeletti S, Battistoni F, Fioravanti M, Bernardini S, Dicuonzo G (2013). Procalcitonin and mid-regional pro-adrenomedullin test combination in sepsis diagnosis. Clin Chem Lab Med.

[23] Mitaka C (2005). Clinical laboratory differentiation of infectious versus non-infectious systemic inflammatory response syndrome. Clin Chim Acta.

[24] Angus DC, van der Poll T (2013). Severe sepsis and septic shock. N Engl J Med.

[25] Salciccioli JD, Marshall DC, Pimentel MA, Santos MD, Pollard T, Celi LA (2015). The association between the neutrophil-to-lymphocyte ratio and mortality in critical illness:an observational cohort study. Crit Care.

[26] Singer M, Deutschman CS, Seymour CW, Shankar-Hari M, Annane D, Bauer M (2016). The Third International Consensus Definitions for Sepsis and Septic Shock (Sepsis-3). JAMA.

